# On Encapsulated Dielectric Barrier Discharge Plasma Sources for Radar Cross Section Reduction in Mobile Environments

**DOI:** 10.3390/s23229170

**Published:** 2023-11-14

**Authors:** Minsu Choi, Shin-Jae You, Jinwoo Jung, Changseok Cho, Yongshik Lee, Cheonyoung Kim, Jungje Ha, Hyunsoo Lee, Youbin Seol

**Affiliations:** 1 Department of Physics, Chungnam National University, Daejeon 34134, Republic of Korea; bss125576@naver.com (M.C.); sjyou@cnu.ac.kr (S.-J.Y.); 2 Institute of Quantum Systems (IQS), Chungnam National University, Daejeon 34134, Republic of Korea; 3 Department of Electrical and Electronic Engineering, Yonsei University, Seoul 03722, Republic of Korea; jjw1805@yonsei.ac.kr (J.J.); changseok.cho@yonsei.ac.kr (C.C.); yongshik.lee@yonsei.ac.kr (Y.L.); 4 Agency for Defense Development (ADD), Daejeon 34186, Republic of Korea; cykim93@add.re.kr (C.K.); jungjeha@add.re.kr (J.H.); hslee83@add.re.kr (H.L.)

**Keywords:** Dielectric Barrier Discharge (DBD), Radar Cross Section reduction, Lissajous figure

## Abstract

This paper deals with the practical application of Radar Cross Section (RCS) reduction technology using plasma. Although various plasma application technologies for RCS reduction have been studied, there are still many issues to be addressed for practical implementation. In order to achieve actual application, the discharge should be sustained regardless of the external environment of the aircraft. It is also important to investigate the actual plasma parameters to determine the expected RCS reduction effect. Building upon previous studies that optimized the electrodes for RCS reduction, this study fabricates a Dielectric Barrier Discharge (DBD) source suitable for dynamic environments and verifies the power consumption during one cycle of plasma generation. The obtained results are expected to contribute to the optimization of DBD electrodes for plasma RCS reduction.

## 1. Introduction

Radar Cross Section (RCS) reduction technology in modern warfare is a cutting-edge field being extensively researched by many advanced countries as a crucial technology directly related to the survival of aircraft. Among various RCS reduction techniques, the use of plasma has been extensively studied, leading the development of aircraft stealth technology. RCS using plasma is based on the scattering properties resulting from the behavior of electrons in plasma.

Recently, various methods of utilizing plasma for RCS have been researched. These include developing a compact plasma source with checkerboard patterned electrodes [1,2,3], creating a specific plasma source in the form of fluorescent lamps to verify the reduction effect [4,5], and designing plasma configurations to encompass jet engines that emit aircraft gases [6,7,8]. Furthermore, extensive research is being conducted on plasma sources, including the development of flexible electrodes as plasma generation elements in board form, for applications in aircrafts [9,10,11].

One of the more effective methods for reducing RCS using plasma is Dielectric Barrier Discharge (DBD). DBD provides the advantage of a low breakdown voltage [12] compared to other discharge sources, making it suitable for a wide range of applications, including RCS reduction, industrial processing, and even in the field of plasma medicine [13,14,15,16,17,18]. The basic structure of DBD typically involves two opposing electrode plates where one or both electrodes are covered by dielectrics. Y.S. Lee et al. applied variations to this basic design by selectively covering one side with a dielectric and confirmed RCS reduction effects with different electrode configurations in the dielectric-covered region [14,15,16]. However, there are still several challenges in applying these results in practical applications.

The first challenge is that a DBD source for RCS reduction requires the optimization of electrode discharge regions or independent control of its internal and external environments. The aircraft operates at slightly lower pressures (0.3 atm) than atmospheric pressure and moves at high speeds approaching Mach numbers, necessitating that the discharge remains unaffected by the high velocity.

The second challenge is the difficulty in plasma diagnostics. Currently used DBD sources have temporal and spatial non-uniformities of the current and it is difficult to determine the plasma parameters [19]. Therefore, it is necessary to analyze the DBD source using reliable methods to understand the actual plasma parameters and predict the future results for RCS reduction.

In this paper, for independent control in gas environments and high-speed flow, several types of encapsulated DBD plasma sources sealed by an acrylic skeleton are introduced. It is confirmed that the discharge behavior remains relatively stable over time, even when discharge is conducted independently. As a DBD plasma diagnostics method, Lissajous figures, the well-known volt-coulomb (V-Q) characteristic [19,20,21,22], is employed to examine the variation trends of power values with respect to voltage and frequency for the three different sources.

## 2. Materials and Methods

(a) DBD source structure and discharge characteristics

**Figure 1:**
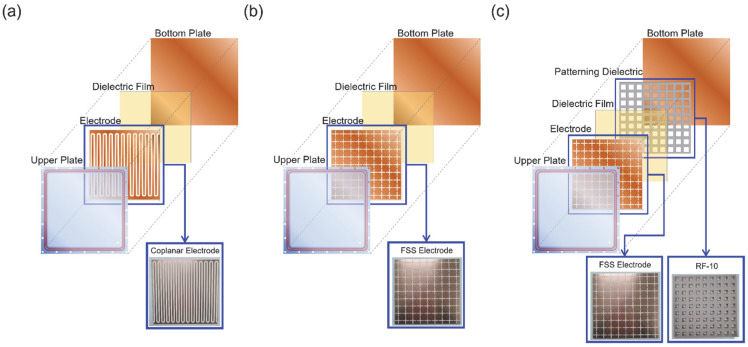
The Dielectric Barrier Discharge (DBD) plasma source employed in the experiment had the following electrode configurations: (**a**) Coplanar electrode configuration, (**b**) frequency selective surface (FSS) electrode configuration, and (**c**) FSS electrode configuration with a patterned dielectric layer RF-10.

The DBD plasma generator, as depicted in Figure 1, utilizes an upper plate made of acrylic material measuring $200\times 200\times 10\;{mm}^{3}$ to isolate from the external environment. A square-shaped pit measuring $160\times 160\times 5\;{mm}^{3}$ is created on one side of the acrylic plate to establish a discharge region within. The electrode is inserted into the pit and is covered by a 70 μm polyimide dielectric film. The electrode attached to the upper plate is connected to a wire passing through the acrylic, and it is bonded to the acrylic plate using a sealing bond (940LE, SANUL CO., LTD, Seoul, Republic of Korea). The upper plate is then combined with the bottom plate, and the internal gas conditions are isolated from the external gas conditions by the presence of dual silicon O-rings in the side. Various types of electrodes are adopted for the characterization of plasmas. Each electrode type has already been proved to show an RCS reduction effect. Figure 1a shows a coplanar electrode (COP) using the same structure [14] and (b) shows a frequency selective surface (FSS) electrode, as conducted in experiments by Y.S. Lee et al. [15]. Therefore, in cases (a) and (b), the plasma is discharged between the polyimide dielectric barrier and the bottom plate. In case (c), a patterned dielectric is added. The introduction of patterned dielectric material can lead to an increase in the real part of the effective permittivity between the electrodes, potentially enhancing the attenuation effect [16]. In this case, plasma is generated between the polyimide film and the patterned dielectric (FSS + Patterning).

(b) DBD source voltage measurement

**Figure 2:**
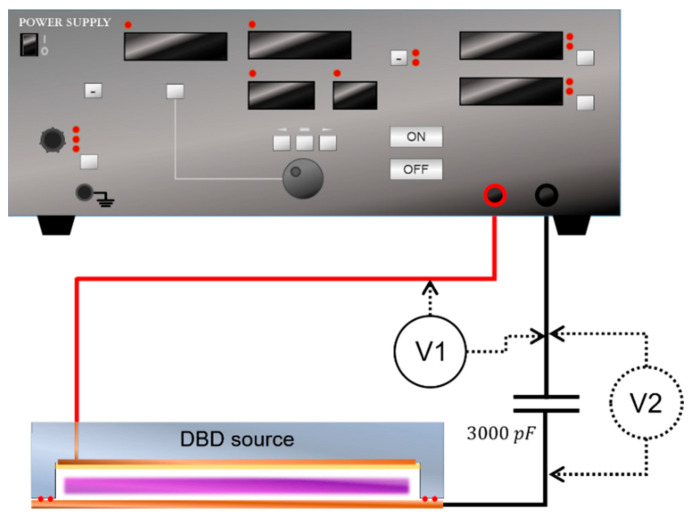
A schematic design of the experimental setup. Voltage probe 1 measures the overall system voltage, while voltage probe 2 measures the voltage across a 3000 pF test capacitor.

The schematic design of the experiment is shown in Figure 2. The internal pressure of the DBD source is fixed at 1 Torr of argon gas and the external condition is at atmospheric pressure. A square wave with a driving voltage of 1.5–2.5 kV and frequency of 2–10 kHz is delivered from a power generator. A system voltage probe (P5100, Tektronix, Beaverton, OR, USA) is used for the voltage measurement. To measure the current, a test capacitor of 3000 pF is connected to the DBD source with a voltage probe (P5100A, Tektronix, United States). The measured data from both probes are transmitted to a single oscilloscope (TDS 3054C, Tektronix, Beaverton, OR, USA). All measurements are taken within 1 min after the initiation of DBD source discharge. For better validation, voltage measurements are performed five times for each plasma source.

(c) Methods

**Figure 3:**
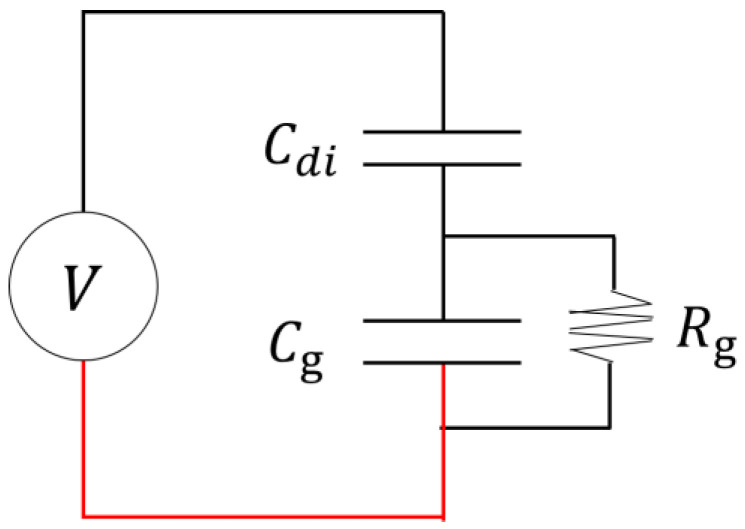
Equivalent circuit model of the dielectric barrier discharge plasma source.

The voltage measurements are used to investigate the voltage applied to the DBD source and the charge amount flowing through the wire from the additional capacitor over time. The power consumption of the DBD source in one cycle is examined under various frequencies and voltages. According to the Drude model, the plasma frequency is related to the RCS reduction effect and depends on the electron density [23,24]. When conducting RCS reduction studies, it is important to measure the plasma electron density. However, there is a significant limitation in the current DBD source, which prevents direct insertion of a probe into the plasma. Therefore, in this study, the power consumed by the DBD source in one cycle is investigated using the measured voltage under different frequencies and voltages. It was reported that the plasma density is directly related to the input power as the density commonly increases with the power [25,26]. The equivalent circuit model of this study is shown in Figure 3.

The equivalent circuit of the DBD source can be expressed as Equation (1) based on Kirchhoff’s voltage law.

(1) $$V_{g}=V-V_{di}$$

$V_{g}$ represents the electrode gap voltage, V represents the measured voltage of the entire DBD plasma generator system, and $V_{di}$ represents the voltage applied to the dielectric barrier (polyimide) within the DBD source. In Equation (1), the values of V can be obtained through measurements. However, $V_{di}$ can be solved using Equation (2). As for $C_{di}$, since it is fabricated in a form that covers the entire electrode, it is calculated using the parallel plate capacitor model.

(2) $$V_{di}=\frac{Q_{di}}{C_{di}}$$

The value of $Q_{di}$ is determined by the current.

(3) $$Q_{di}=\int Idt$$

Subsequently, considering the sum of $V_{g}$ and $V_{di}$ as the voltage across the DBD source, it can be expressed as Equation (4).

(4) $$V_{g}\left(t\right)+V_{di}(t)=V_{DBD}\left(t\right)$$

Furthermore, the rate of change of $V_{g}$ and $V_{di}$ with respect to time can be written as Equations (5) and (6), respectively.

(5) $$\frac{{dV}_{g}\left(t\right)}{dt}=\frac{1}{C_{g}}\left(I\left(t\right)-I_{a}\left(t\right)\right)$$

(6) $$\frac{{dV}_{di}\left(t\right)}{dt}=\frac{I\left(t\right)}{C_{di}}$$

By substituting Equations (5) and (6) into Equation (4), Equation (7) is obtained as

(7) $$I_{a}\left(t\right)=I\left(t\right)\frac{C_{g}+C_{di}}{C_{di}}-C_{g}\frac{\partial U}{\partial t}$$

According to the Q-V characteristics, when plotting the Lissajous figures for one cycle, it takes the form of a trapezoid [19,20,21,22]. The shape and slope of the trapezoid are determined by various variables. Additionally, by calculating the width of the trapezoid, we can determine the energy consumed during one cycle. The energy of one cycle (E) determined by $∆V_{g}$ and $∆Q_{g}$ is expressed in Equation (8).

(8) $$E=∆V_{g}\times ∆Q_{g}$$

Afterwards, the energy is divided by the duration of one cycle (T) to calculate the average power [27].

(9) $$P=\frac{1}{T}E$$

## 3. Results and Discussion

**Figure 4:**
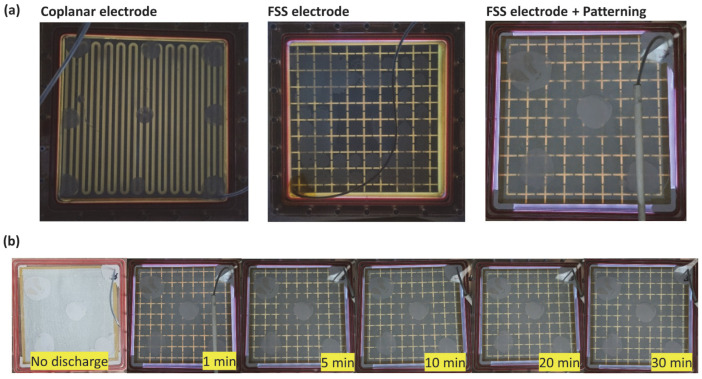
Discharge appearance of the FSS plasma generator: (**a**) appearance immediately after discharge from each electrode, and (**b**) temporal evolution of the FSS electrode. The photo was taken on top of the DBD source and appears as yellow light due to the polyimide dielectric.

Figure 4a shows the discharge images of the encapsulated DBD sources with different electrode structures. Under atmospheric conditions, the three types of DBD source exhibit uniform discharge across all electrodes inside the source. Light emitted from the uncovered cross-shaped region, not shielded by the electrodes, shows the discharge. As in Figure 4b, it is confirmed that the discharge is sustained stably for a duration of 30 min. The COP and FSS structures manifest as yellowish, which is attributed to the presence of the polyimide film. Conversely, in the case of FSS + Patterning, a violet hue is discerned due to the absence of polyimide film on the lateral surfaces.

**Figure 5:**
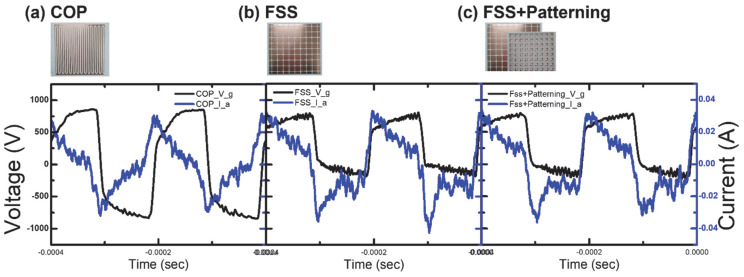
The voltage–current graph of the DBD (Dielectric Barrier Discharge) calculated as described in the paper, using the provided data for $V_{g}$ (voltage across the plasma resistance $R_{g}$) and $I_{a}$ (current flowing in $R_{g}$). The presented results correspond to a driving voltage of 2.5 kV and a driving frequency of 5 kHz.

The voltage–current results obtained from the three DBD sources are presented in Figure 5. Voltage data are collected within 1 min after the initiation of the discharge, and the measurements are repeated 5 times to ensure accuracy. In Figure 5, $V_{g}$ and $I_{a}$ represent the voltage across the plasma resistance $R_{g}$ and the current flowing through $R_{g}$ from the measured data, respectively. Notably, the current results exhibit similarities in the observed trends of effective DBD discharge under the same square-wave generator power conditions [13,28]. Overall, the trends observed in the FSS and FSS + Patterning configurations are similar, while the Coplanar configuration shows slightly different results. The patterned dielectric does not affect the voltage–current shape and the difference can be attributed to variations in the electrode’s shape as the dielectric thickness remains the same. FSS-based structures have slightly biased and noisy voltage profiles. This shows that the shape of electrodes directly affects the circuit characteristics and plasma operations even in the similar structures. Additionally, a notable divergence is observed between the applied voltage and the voltage across the electrodes. This phenomenon is consistent with experiments exhibiting DBD characteristics in other instances, but the underlying cause remains elusive [13,15].

**Figure 6:**
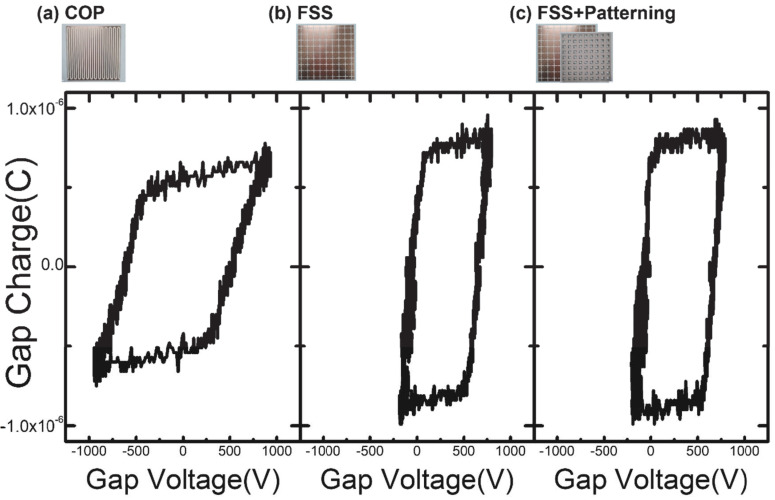
Lissajous figure, the Q-V (Charge–Voltage) diagram for each DBD source. The presented graphs depict the results for one complete cycle. The presented results correspond to a driving voltage of 2.5 kV and a driving frequency of 5 kHz.

For the detailed analysis, Lissajous figures of the three types of DBD source with different electrode configurations are derived as in Figure 6. The area under the Q-V graph represents the energy consumed during one cycle [20]. All configurations display a trapezoid. Similar to the voltage–current data in Figure 5, the shape of the Q-V graph of the FSS and FSS + Patterning configurations are similar, while that of the Coplanar configuration shows some differences. In the case of FSS and FSS + Patterning, despite having lower applied voltages compared to the coplanar electrode structure, it can be observed that they possess higher charge values. Having a greater charge when the same voltage is applied signifies a larger capacitance. This observation can be explained by the FSS electrode and the FSS + Patterning electrode having a larger electrode area compared to the Coplanar structure or conventional DBD plasma sources, resulting in a greater capacitance.

This result indicates that the variation in capacitance due to the electrode structure has a greater impact than the presence or absence of dielectric material in the middle.

**Figure 7:**
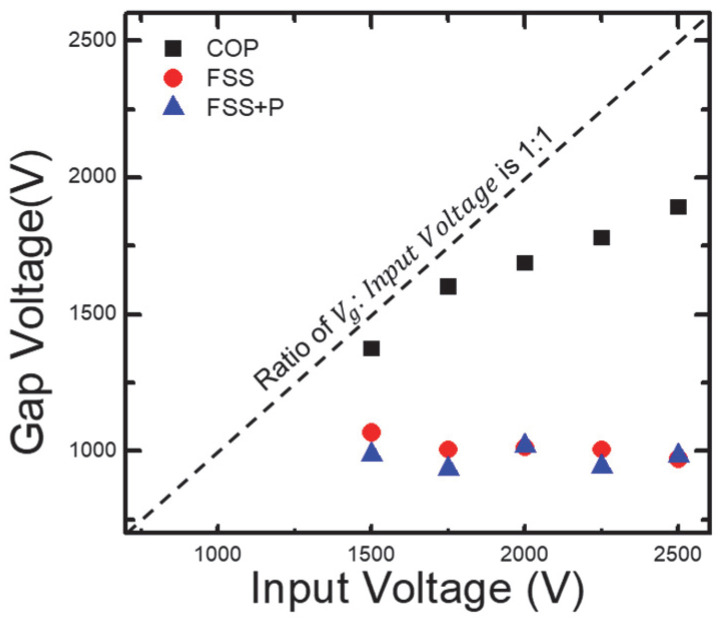
A plot of the ratio of the gap voltage to the input voltage. The black square dots represent the case of a coplanar structure, the red circles represent the form of FSS electrodes, and the blue triangles represent the case where patterned dielectrics are added.

Figure 7 shows the applied voltage and the gap voltage between the applied voltage and the DBD source. In the case of the coplanar structure, the gap voltage increases with the applied voltage. This result indicates that the coplanar structure exhibits efficient voltage control. On the other hand, for the FSS and FSS + Patterning structures, it can be observed that the gap voltage remains constant regardless of the applied voltage. In the context of our research, this suggests that the sensitivity of the operating voltage to the shape of the FSS electrode is lower than that of the Coplanar electrode, irrespective of the presence of the patterning dielectric.

**Figure 8:**
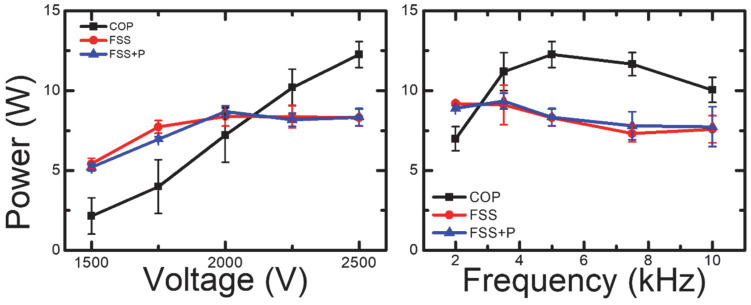
The power change according to the driving frequency and driving voltage of 3 types of sources derived from Lissajous figures.

From Lissajous figures, the power consumptions of each type of the source are derived. Figure 8 shows the power consumption depending on input voltage and frequency, respectively. Again, the Coplanar structure shows different behaviors comparing to the cases of the FSS and FSS + Patterning structures. In the case of the Coplanar configuration, the power consumption continues to increase with voltage. On the other hand, the FSS and FSS + Patterning structures exhibit a slight increase in power consumption up to 2000 V, after which it remains relatively constant. Additionally, the variations with driving frequency show that the Coplanar configuration reaches its maximum power consumption at 5 kHz. However, the FSS and FSS + Patterning structures exhibit a slight decrease in power consumption on input frequency. Overall, the Coplanar structure shows higher plasma activation rate in the higher voltage and frequency range compared to FSS-based structures. Also, FSS-based structures show almost constant behaviors on input parameters. These results indicate that the optimal points in power and frequency vary depending on the electrode spacing and shape.

Combining the voltage and power characteristics in Figure 7 and Figure 8, in the case of the coplanar structure, the gap voltage increases along with the applied voltage. Consequently, the power consumed during one cycle also increases. On the contrary, FSS-based structures do not show significant changes in the gap voltage and power with respect to the applied voltage, which can be interpreted as low efficiency in voltage control. Overall, Coplanar structures show higher power coupling to the input signal compared to FSS-based structures. These results give the Coplanar structure the potential of characterization with its controllability by the input signals, whereas FFS-based structures show stable behaviors in wide operation conditions. In the case of the FSS + Patterning, as the patterned dielectric which has already been reported to show additional RCS reduction effects [16] does not have any meaningful physical disruption, we can enhance the RCS reduction effects of the FSS structure without side effects.

## 4. Conclusions

We have simplified the existing DBD source for practical application and developed a DBD source suitable for mobile environments. Three types of DBD sources with different electrode designs are introduced, which are already confirmed to have RCS reduction effects. The fabricated DBD source enables uniform discharge without the need for a separate vacuum system, maintaining consistent discharge characteristics for approximately 30 min. Basic plasma parameters with input signals are analyzed.

As we have compared three types of electrode design, ultimately the shape of electrodes directly affects the circuit characteristics and plasma operations. It is mainly from the different capacitance in different effective electrode areas, while the patterned dielectric does not have any meaningful effect. Overall, the Coplanar structure shows higher controllability by the input signals, whereas FFS-based structures show stable behaviors in wide operating conditions. Focusing on those different characteristics, the characterization of a DBD source for RCS reduction is possible, choosing a proper electrode design. As a basic parameter analysis, these results are beneficial in understanding the variations associated with electrode configuration and the presence of additional dielectrics when designing DBD plasma generators for RCS reduction.

## Data Availability

The data presented in this study are available in the article.
